# Optimized ultrasound-assisted extraction of phenolic compounds from *Thymus comosus* Heuff. ex Griseb. et Schenk (wild thyme) and their bioactive potential^☆^

**DOI:** 10.1016/j.ultsonch.2022.105954

**Published:** 2022-02-16

**Authors:** Mihai Babotă, Oleg Frumuzachi, Alexandru Gâvan, Cristian Iacoviță, José Pinela, Lillian Barros, Isabel C.F.R. Ferreira, Leilei Zhang, Luigi Lucini, Gabriele Rocchetti, Corneliu Tanase, Gianina Crișan, Andrei Mocan

**Affiliations:** a Department of Pharmaceutical Botany, “Iuliu Hațieganu” University of Medicine and Pharmacy, Gheorghe Marinescu Street 23, 400337 Cluj-Napoca, Romania; b Department of Medical Devices, “Iuliu Hațieganu” University of Medicine and Pharmacy, 4 Louis Pasteur, 400349 Cluj-Napoca, Romania; c Department of Pharmaceutical Physics-Biophysics, “Iuliu Hațieganu” University of Medicine and Pharmacy, Louis Pasteur Street 6, 400349 Cluj-Napoca, Romania; d Centro de Investigação de Montanha (CIMO), Instituto Politécnico de Bragança, Campus de Santa Apolónia, 5300-253 Bragança, Portugal; e Department for Sustainable Food Process, Università Cattolica del Sacro Cuore, Via Emilia Parmense 84, 29122 Piacenza, Italy; f Department of Animal Science, Food and Nutrition, Università Cattolica del Sacro Cuore, Via Emilia Parmense 84, 29122 Piacenza, Italy; g Department of Pharmaceutical Botany, “George Emil Palade” University of Medicine, Pharmacy, Sciences and Technology of Târgu Mures, 38 Gheorghe Marinescu Street, 540139 Târgu Mures, Romania; h Laboratory of Chromatography, Institute of Advanced Horticulture Research of Transylvania, University of Agricultural Sciences and Veterinary Medicine, 400372 Cluj-Napoca, Romania

**Keywords:** *Thymus comosus* Heuff. ex Griseb. et Schenk, Ultrasound-assisted extraction, Optimisation, Design of Experiments, Phenolic compounds

## Abstract

An optimized ultrasound-assisted extractive method was developed to obtain a polyphenol-enriched extract from the aerial parts of *Thymus comosus* Heuff. ex Griseb. et Schenk. Optimization process was conducted based on Design of Experiment (DoE) principles, determining the influence of three independent variables (time, ultrasound amplitude, ethanol concentration) on the total phenolic content of the extract (dependent variable). Additionally, the phenolic composition of the extract was characterized through UHPLC-HRMS, revealing beside the most abundant flavonoid-type compounds the presence of salvianolic acids C, D and L in high amounts. Phytochemical profile of the extract was correlated with its antioxidant activity (tested through five complementary assays) and enzyme-inhibitory potential, showing important antiglucosidase and anticholinesterase effects. Overall, it was concluded that the developed method is suitable for obtaining a good recovery of both phenolic and non-phenolic compounds from *Thymus comosus* aerial parts, and their presence in the optimized extract is responsible for its pharmacological potential.

## Highlights

• *T. comosus* is an endemic, less-studied thyme species used in Romanian folk medicine.
• Optimized ultrasound-assisted extraction was applied for the recovery of its bioactive compounds.
• The influence of extraction parameters on the quality of the extract was studied.
• The presence of polyphenols was correlated with bioactive potential of the extract.

## Graphical abstract

**Figure:**
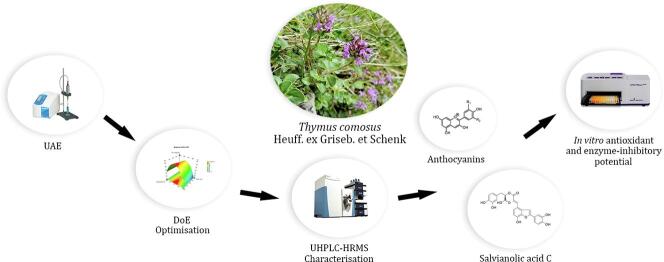

## 1 Introduction

**Fig. 1:**
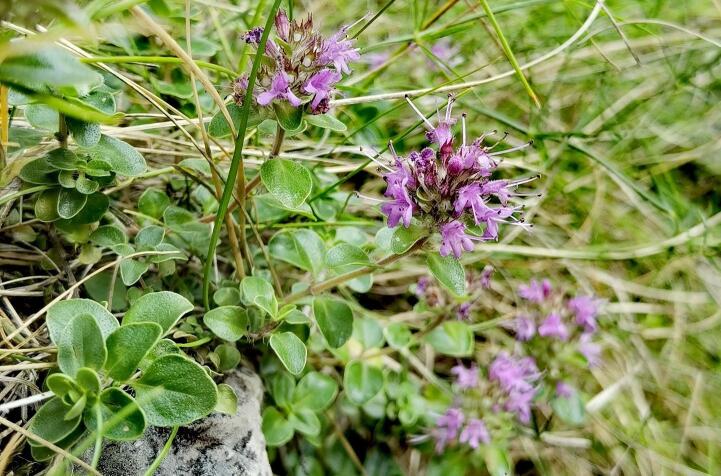
*Thymus comosus* Heuff. ex Griseb. et Schenk.

*Thymus* genus is one of the most representative taxonomic subunity of the Lamiaceae family, comprising more than 215 species distributed worldwide [1], [2]. Different thyme species (i.e. *Thymus vulgaris*, *T. zygis*, *T. serpyllum*) are well-established herbal medicines (having monographs in official pharmacopoeias like European Pharmacopoeia), whose therapeutic potential correlates well with both the high content of volatile oils and the complexity of non-volatile fractions, rich in phenolic acids, flavonoids and iridoids [3], [4]. Moreover, *T. vulgaris* aerial parts (known as *Thymi herba*) are considered one of the most popular plant products used as a spice in household and industrial food applications [2]. Beside the common-used thyme species, there are also reported data about the health-related benefits of other *Thymus* representants.However, their potential applications are underevaluated because their specific distribution (most of them are endemic species) and the small amount of data previously published on their chemical or bioactive features are poorly documented. One of these species is *T. comosus* Heuff. ex Griseb. et Schenk (TC) (Fig. 1) an endemic member of the thyme family that grows exclusively in the Intra-Carpathic regions of Romania. The state-of-the-art regarding its chemical profile and bioactive potential is weakly documented; Boz *et al.* and Pavel *et al.* focused their studies on the characterization of essential oils obtained from its aerial parts, while the phenolic profile of the methanolic extract was studied by Mărculescu *et al.*, revealing the presence of phenolic acids and hydroxycinnamic derivatives in higher amounts than in *T. vulgaris* [5], [6], [7].

In the last decades, a high number of studies were focused on reconsideration of thyme (including some less-studied species belonging to this genus) and their potential use as a source of bioactive compounds with wide applications as medicines and food additives [8], [9], [10], highlighting the importance of enhancing the recovery of bioactive compounds from these species. This goal was mainly achieved using advanced extractive technologies, applied both to improve extraction yields of classical methods or, sometimes, to completely replace them (bringing into account their weak points as long extraction times, big solvents volumes and their toxicity, degradation, and loss of physicochemical/biochemical properties of extracted compounds) [11], [12]. One of the most exploited modern extraction techniques is ultrasound-assisted extraction (UAE), intensively used and studied in last years for its many advantages; it allows to obtain high extraction yields with minimum damage of structural and molecular properties of interest compounds, reduces solvents use, and assures a better penetration of the extracted matrices through specific processes [12], [13], [14]. Acoustic cavitation is the main process involved in ultrasound-assisted extraction, inducing secondary modifications in plant material through fragmentation, localised erosion, pore formation or shear forces. All of these factors enhance the disruption of cell walls, enhancing the contact of extractible compounds with the solvent and their partition in the extraction environment [14]. It was also shown that the UAE could be more effective when the influence of each extraction parameter is well defined and correlated with the properties of the extracted matrix, these features being overall reflected by the final quality of the obtained extracts in terms of their bioactive potential [12], [15], [16].

Several statistic and computational methods were applied to optimize different UAE methods, including Design of Experiments (DoE) [17], [18], [19], [20]. Methods that are conventionally used for optimisation purposes, where the influence of one factor is studied at a time, present a major shortcoming. They do not consider potential interactions between factors, which can mislead in identifying the optimal combination of critical process parameters [21], [22], [23]. The recently alternative of using the Design of Experiments (DoE) to find optimal product and process characteristics provides the highest amount of information from the least number of experimental runs by systematic variation of the factors and simultaneously evaluation of the effects of multiple variables. The design space is a well-defined multidimensional range of input variables and/or process parameters demonstrated to ensure that the obtained product would meet predefined specifications [24], [25].

Considering the above, the present study aimed to develop an optimized ultrasound-assisted extraction method to obtain a polyphenol-enriched extract from aerial parts of TC based on DoE principles. Additionally, the phenolic profile of the optimized extract was analyzed through a chromatographic method, while antioxidant and enzyme-inhibitory properties were tested using *in vitro* assays. The primary outcome of our work was to emphasize the positive impact of the optimized UAE method on the final quality of the extract and also to bring a new perspective regarding the potential use of this less-studied *Thymus* species as a valuable source of bioactive compounds.

## 2 Experimental setup

### 2.1 Plant material

In this work, *T. comosus* Heuff. ex Griseb. et Schenk aerial parts were collected in august 2019 from Rimetea (Alba county, Romania) during their maximum flowering period. Plant material was sorted, supposed to authentication procedure based on its botanical features, and dried at room temperature in a shady place until a constant mass; dried material was kept in paper bags in the herbarium of Pharmaceutical Botany Department of Iuliu Hațieganu University of Medicine and Pharmacy Cluj-Napoca until extraction.

### 2.2 Extraction procedure

Dried plant material was previously powdered using a laboratory mill (Grindomix® GM 200, Retsch Gmbh., Germany) and manually sieved (1 mm standard sieve according to PhEur 10.6) to ensure particles' uniformity. UAE was carried out using a Vibra-Cell™ Ultrasonic Processor, model VCX 500 equipped with a tapered microtip (ø 6 mm) and integrated temperature controller. In order to generate a D-optimal experimental design for the optimization of the extraction process, three parameters were taken into account as independent process variables: exposure time (1, 3, 5, 7, and 10 min), ultrasounds amplitude (20, 30, and 40%) and ethanolic concentration of the solvent (30, 50, and 70%). Practically, for each experimental run, 2 g of TC powder were mixed with 20 mL of solvent and supposed to be extracted under an ice bath to avoid heating. The mixture was further vacuum filtered for the separation of supernatant. After establishing optimal extraction parameters, the optimized *T. comosus* extract (OpTC) was freeze-dried and kept in a desiccator at room temperature until phytochemical and bioactivity assays.

### 2.3 Design of experiments

The MODDE 12.1 software (Sartorius Stedim, Sweden) was used to develop the design of experiments (DoE), which allowed the introduction of the experimental variability to study its effects and establish the optimal experimental values for the extraction purpose.

The DoE type was D-optimal, which is based on the selection of experimental runs to span the largest possible volume of the variability matrix. This kind of model was specially created to allow the study of multiple combinations of qualitative and quantitative multilevel factors in the same experimental design [26]. The analysis of coefficients evaluated the effects of the process variables over the measured extraction performances. Based on the obtained results, the optimal formulation and design space were further defined.

Moreover, a Proven Acceptable Range (PAR) was established within the design space, which is graphically represented by a design space hypercube, marking the largest possible regular surface that can be inserted into the irregular design space and showing the volume in which all factor combinations can be used without compromising the critical quality attributes of the end product [27]. After determining the design space and PAR, the optimal formulation was established by defining combinations of factor values that predict a result as close as possible to the target values of the response [28], [29].

### 2.4 Total phenolic content (TPC) and total flavonoidic content (TFC)

The protocol used for TPC determination was based on the Folin-Ciocalteu method adapted to the microplate reader, previously reported by Babotă et al. [30]. The absorbance of samples was read at 760 nm after 30 min incubation at room temperature, results being expressed as milligrams of gallic acid equivalents per milliliter of raw extract (for the samples resulting from experimental runs of optimization process (mg GAE/mL); for the optimized extract, results were expressed per g of dried extract (mg GAE/g). TFC was determined using the aluminium chloride method based on the protocol described by the same authors [30], results being expressed as milligrams of rutin equivalents (mg RE/g extract).

### 2.5 UHPLC-HRMS analysis of phenolic profile

The optimized *T. comosus* dried extract (100 mg) was dissolved in 2 mL of ethanol 50% and centrifuged at 6000×*g* for 10 min at 4 °C. After that, the supernatant was transferred to HPLC vials for instrumental analysis. The untargeted phenolic profiling was carried out by high-resolution mass spectrometry (HRMS) on a Q-Exactive™ Focus Hybrid Quadrupole-Orbitrap Mass Spectrometer (Thermo Scientific, Waltham, MA, USA) coupled to a Vanquish ultra-high-pressure liquid chromatography (UHPLC) pump and equipped with heated electrospray ionization (HESI)-II probe (Thermo Scientific, USA). The chromatographic separation was achieved under a water-acetonitrile (both LC-MS grade, from Sigma-Aldrich, Milan, Italy) gradient elution (6–94% acetonitrile in 35 min, adding 0.1% formic acid to both phases) on an Agilent Zorbax Eclipse Plus C18 column (50 × 2.1 mm, 1.8 μm). The HRMS conditions were adapted from a previously published work [31]. The flow rate was 200 μL/min, the injection volume was 6 μL, using a full scan MS-data-dependent (Top N = 3) MS/MS mode. In the full scan mode, the acquisition was achieved in the range 100–1200 *m*/*z*, with a positive ionization mode and a mass resolution of 70,000 FWHM. The automatic gain control target (AGC target) and the maximum injection time (IT) of the Orbitrap were 1e^6^ and 200 ms, respectively. In the data-dependent MS/MS mode, the full scan mass resolution was reduced to 17,500 at *m*/*z* 200, with an AGC target value of 1e^5^, maximum IT of 100 ms, and isolation window of 1.0 *m*/*z*, respectively. The Top N ions were selected for fragmentation using 10, 20, 40 eV normalized collisional energies. The HESI parameters are reported in previous work [32]. The raw data (.RAW files) were further processed using the software MS-DIAL (version 4.70) for post-acquisition data filtering [33], and the annotation was done via spectral matching against the databases FoodDB and Phenol-Explorer. The identification step was based on mass accuracy (setting a 5-ppm tolerance for *m*/*z* values), isotopic pattern, and spectral matching. These criteria were used to calculate a total identification score, considering the most common HESI+ adducts for the chromatographic conditions adopted, thus reaching a level 2 of confidence in annotation [34]. Finally, the cumulative intensity values of the different phenolic classes annotated were converted into semi-quantitative data, exploiting hydroalcholic standard solutions of pure compounds (Extrasynthese, Lyon, France) analyzed under the same conditions. In this regard, ferulic acid (phenolic acids), quercetin (flavonols), catechin (flavanols), cyanidin (anthocyanins), luteolin (flavones and other flavonoids), resveratrol (stilbenes), and oleuropein (other remaining phenolics) were used as representatives of their respective classes. In this regard, a linear fitting (R^2^ greater than 0.99) was built and used for quantification, and results were expressed as mg equivalents (Eq.)/g lyophilized extract (n = 3).

### 2.6 In vitro biochemical assay of antioxidant potential

Five complementary assays were used to evaluate the antioxidant potential of OpTC through different pathways: DPPH, TEAC (indicating radical scavenger activity), FRAP (ferric reducing antioxidant power), TBARS (evaluating lipid peroxidation through thiobarbituric acid reactive substances formation inhibition), and OxHLIA (oxidative haemolysis inhibition assay). For all assays, the detailed protocols are extensively described in our previous works [30], [35], [36], [37], [38].

In DPPH assay, 30 μL of sample (1 mg/mL) were mixed with 270 μL of 0.004% methanol solution of DPPH. The absorbance was read at 517 nm after a 30 min incubation at room temperature in the dark. DPPH radical scavenging activity was expressed as milligrams of Trolox equivalents (mg TE/g extract) [30], [36].

For TEAC assay, ABTS^+^ radical solution was previously prepared by reacting 7 mM ABTS solution with 2.45 mM potassium persulfate and allowing the mixture to stand for 12–16 in the dark at room temperature. Prior to beginning the assay, ABTS^+^ solution was diluted with distilled water to an absorbance of 0.70 ± 0.02 at 734 nm; 200 μL of radical solution were added to 20 μL of the sample (1 mg/mL) and incubated for 30 min at room temperature. The absorbance was read after incubation at 734 nm, radical scavenging activity being expressed as milligrams of Trolox equivalents (mg TE/g extract) [30], [36].

Ferric reducing antioxidant power (FRAP) was tested using the premixed FRAP reagent: acetate buffer (0.3 M, pH 3.6), 2,4,6-tris(2-pyridyl)-*S*-triazine (TPTZ) (10 mM) in 40 mM HCl and ferric chloride (20 mM) in a ratio of 10:1:1 (*v/v/v*). Samples were reconstituted at 1 mg/mL; the absorbance was read at 593 nm after a 30 min incubation at room temperature, FRAP activity being expressed as milligrams of Trolox equivalents (mg TE/g extract) [30], [36].

For the evaluation of lipid peroxidation inhibitory activity through TBARS assay, the extract was submitted to serial dilutions; 200 μL from each dilution were mixed in an Eppendorff reaction tube (2 mL) with 100 μL of FeSO_4_ (10 μM) and 100 μL of ascorbic acid (0.1 mM), reaction mix being incubated for 1 h 37 °C. Incubation was followed by adding 500 μL of trichloroacetic acid (28% *w/v*) and 380 μL of thiobarbituric acid (TBA, 2% *w/v*) then heating at 80 °C for 20 min. To quantify malondialdehyde (MDA)-TBA complexes, reaction tubes were centrifuged at 3000×*g* for 10 min, and the absorbance of the supernatant was read at 532 nm. Results were expressed as EC_50_ values (μg/mL) [38].

For OxHLIA assay, an erythrocyte solution (2.8%, *v/v*; 200 µL) prepared in phosphate-buffered saline (PBS, pH 7.4) was mixed with 400 µL of either: extract solution (1.8–60 µg/mL in PBS), PBS (negative control), distilled water (baseline) or trolox (positive control; 7.81–250 µg/mL in PBS). After pre-incubation at 37 °C for 10 min with shaking, 200 μL of 2,2′-azobis(2-methylpropionamidine) dihydrochloride (AAPH; 160 mM in PBS; from Sigma-Aldrich, St. Louis, MO, USA) were added, and the optical density was measured kinetically at 690 nm in an ELx800 microplate reader (Bio-Tek Instruments, Winooski, VT, USA) until complete haemolysis. IC_50_ values (µg/mL) for Δ*t* of 60 and 120 min were obtained by correlating the extract concentration to the Δ*t* values (min), which resulted from the half haemolysis time (H*t*_50_ values) obtained from the haemolytic curves of each extract concentration minus the H*t*_50_ value of the PBS control [37].

### 2.7 Enzyme inhibitory activity

The enzyme-inhibitory ability of OpTC was evaluated against α-glucosidase (α-Glu), tyrosinase acetylcholinesterase using *in vitro* methods. α-Glucosidase inhibition was tested based on a protocol previously described slightly modified [39], 50 μL of extract (1 mg/mL) were mixed with 50 μL of the enzyme (in phosphate buffer, pH 6.8) and 50 μL of the substrate (PNPG, 10 mM in phosphate buffer). The reaction mix was incubated at 37 °C for 15 min, and the absorbance was read at 400 nm using acarbose as a positive control. Results were expressed in terms of IC_50_ (μg/mL).

For tyrosinase (Tyr) inhibition assay, the sample (25 μL, 1 mg/mL) was mixed with tyrosinase solution (40 μL, 10 U/mL) and phosphate buffer (100 μL, pH 6.8), followed by a 15 min incubation at room temperature. After incubation, 40 μL of L-DOPA (2.5 mM in phosphate buffer) were added and the reaction mixture was re-incubated for 10 min in the same conditions. The absorbance was measured at 492 nm and results were expressed as IC_50_ (μg/mL) using as positive control kojic acid [30].

Acetylcholinesterase (AChE) inhibitory activity was tested using an Ellman’s method based protocol. 25 μL of the sample, 50 μL of Tris-HCL buffer (pH 8,50 mM), 125 μL of DTNB (0.9 mM in the same buffer) and 25 μL of AchE (0.078 U/mL in the same buffer) were mixed and incubated for 15 min at room temperature in a dark place. After incubation, 25 μL of ATCI (4.5 mM in Tris-HCl buffer) were added, and the sample was incubated again for 10 min. The absorbance was read at 405 nm, and IC_50_ values (μg/mL) were expressed using galantamine as a positive control [39], [40].

### 2.8 Statistic and correlation analysis

All assays were made in triplicate and the results were expressed as mean ± SD (standard deviation). Finally, correlogram, Pearson's correlation coefficients, and *p*-value matrix (*p* < 0.05), evaluated among different phenolic classes and biological activities, were performed using R-studio software.

## 3 Results and discussion

### 3.1 Design of experiments and experimental model fitting

When both quantitative and qualitative factors are studied in a D-optimal DoE, the selection of experimental runs included in the design matrix is critical and should be performed based on scientific means. In the present case, two statistical parameters were considered: *condition number* and *G-efficiency*. The condition number measures the sphericity of a design, and it is computed for an extended design matrix and represents the ratio of the largest and smallest values of the variability matrix. The ideal condition number value is 1, representing an orthogonal design, the orthogonality of a design evolving inversely proportional with this parameter. For optimization designs with quantitative multilevel factors, as in the present case, the condition number value could increase considerably, and therefore a DoE with a condition number <8 is considered statistically efficient. The G-efficiency is a criterion that expresses the design performance by comparing it to the capacities of a fractional factorial design, expressed as a percentage. For high-quality, reliable D-optimal DoE, a G-efficiency above 60–70% is recommended [26].

**Fig. 2:**
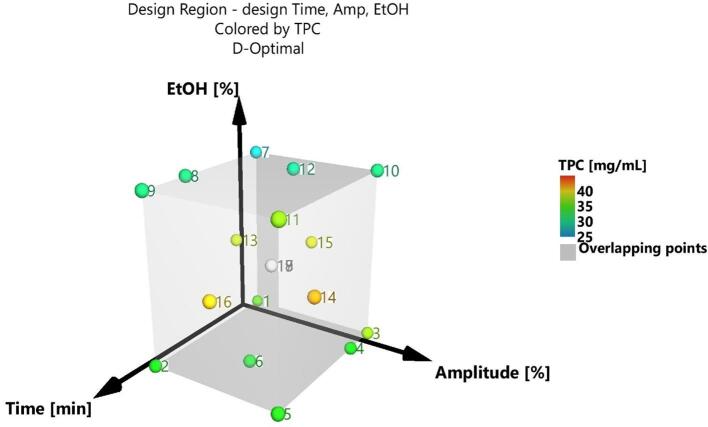
Graphical transposition of the DoE matrix.

The developed optimization DoE took into account three quantitative factors – exposure time (1, 3, 5, 7, and 10 min), ultrasounds amplitude (20, 30, and 40%), and ethanolic concentration of the solvent (30, 50, and 70%) – and consisted in 19 experimental runs, including 3 replicates performed in order to assess the reproducibility of the extractions. To reduce the risk of systematic errors, the 19 runs were performed in a randomized order (Table 1). The DoE registered a condition number of 5.84 and a G-efficiency of 78.26%, which describe a highly reliable statistical model. The studied response was the TPC, extracted for each sample (see Table 1). The graphical transposition of the DoE matrix is presented in Fig. 2.

**Table 1:** DoE matrix and TPC values (mg/mL) of the extracts corresponding to each experimental run.

Exp No	Exp Name	Run Order	Time (min)	Amplitude (%)	EtOH (%)	TPC (mg/mL)
1	N1	18	1	20	30	35.35
2	N2	13	10	20	30	33.48
3	N3	7	1	40	30	37.03
4	N4	15	3	40	30	33.35
5	N5	10	10	40	30	33.78
6	N6	4	7	30	30	32.96
7	N7	11	1	20	70	27.62
8	N8	16	7	20	70	30.52
9	N9	5	10	20	70	30.76
10	N10	17	1	40	70	30.52
11	N11	6	10	40	70	36.84
12	N12	12	3	30	70	29.55
13	N13	3	3	20	50	38.16
14	N14	8	7	40	50	41.34
15	N15	19	1	30	50	38.84
16	N16	1	10	30	50	40.19
17	N17	14	5	30	50	35.72
18	N18	9	5	30	50	40.71
19	N19	2	5	30	50	40.46

The obtained response results were centralized, introduced into the design matrix, and further, the fitting of the experimental data was performed through multiple linear regression (MLR). The model fitting has been evaluated using the standard, most reliable statistical parameters: *R^2^* (goodness of fit), which describes the fraction of the response variation explained by the model, *Q^2^* (goodness of prediction), which estimates the prediction capacities of the model, *ANOVA test* and *model reproducibility* calculated and represented strictly based on the replicates specified in the design matrix. The values that describe each parameter are presented in Table 2. A good fitting is represented by high values of the model performance indicators, as close to value 1 as possible. Moreover, for a valid model, the difference between R^2^ and Q^2^ should be no more than 0.2–0.3 (0081 for our model), as higher differences indicate an inappropriately selected model. Reproducibility should be well over 0.5. The results confirm that the developed model is statistically good, showing a significant influence on the responses and that the model has no lack of fit. Similar statistical results have been registered in other studies where the DoE has been applied for optimization purposes [17], [21].

**Table 2:** The values of parameters used to evaluate experimental model fitting.

	*R^2^*	*R^2^* Adj.	*Q^2^*	*SDY*	*RSD*	*N*	Model Validity	Reproducibility
**TPC**	0.953	0.930	0.872	4.225	1.122	19	0.972	0.846

### 3.2 Process variables effects on the extracted TPC

Based on the DoE model, regression coefficients were automatically established for the studied variables by following the bellow mentioned equation (Eq. (1)):

(1) $$Y_{n}=38.72+0.78X_{1}+1.12X_{2}-1.54X_{3}+1.88X_{1}X_{3}+1.01X_{2}X_{3}-6.72X_{3}^{2}$$

where Y_n_ is the dependent variable; 38.72 is the model constant; 0.78, 1.12, 1.54 represent linear coefficients; 1.88, 1.01 are interaction coefficients between two factors and 6.72 is a quadratic coefficient. The multilevel factor are noted in the equation as X_1_, X_2_, X_3_, representing the exposure time, extraction amplitude and the ethanolic concentration, respectively.

**Fig. 3:**
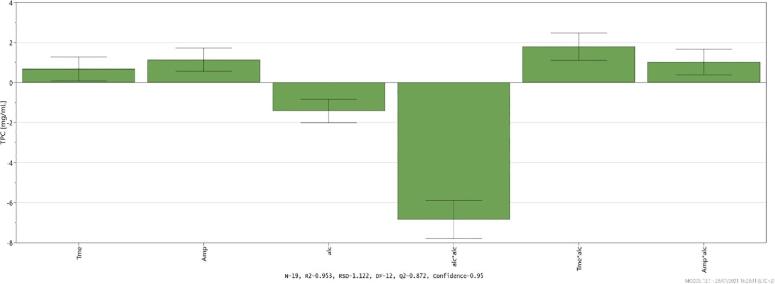
Scaled and centered coefficient plot of the process parameter influence.

**Fig. 4:**
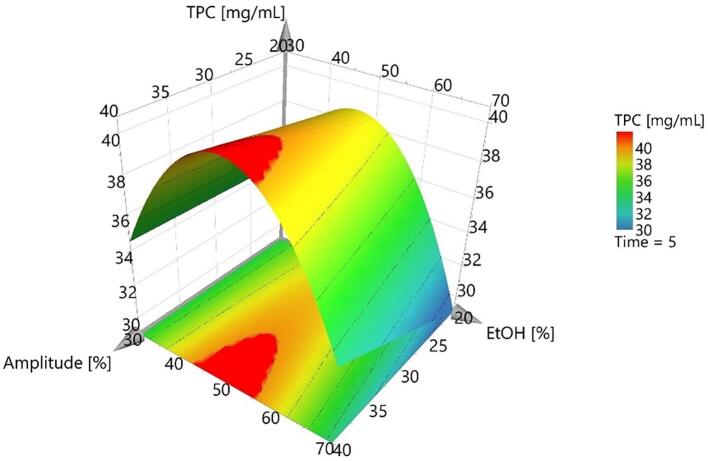
Response surface plot illustrating solvent influence on the UAE developed method.

The equation coefficients aid to quantify the influence of each process parameter (i.e. factor) over the extracted TPC content. A scaled and centered coefficient plot highlighting the factor’s influence is presented in Fig. 3. As we expected, each extraction parameter induced quality changes regarding TPC for the extracts obtained from the experimental runs, additionally the DoE approach allowed us to study the interactions between these parameters and establish which of these interactions had a major influence on TPC. The first parameter analyzed was the solvent, being known that it is the main part of extraction mixtures and its features need to be compatible with the chosen method and extracted compounds [14], [41]. A quadratic interaction was observed regarding the solvent, meaning that the ethanol concentration influence was not linear. This fact is noticeable in the three dimensional response surface plot presented Fig. 4 and can be explained based on the best TPC results obtained using the intermediate ethanol concentration. Moreover, this trend can be correlated with the ability of the mixtures containing intermediate ethanolic concentration to interact with phenolic compounds from TC that are more or less polar to medium-polar compounds (see UHPLC-HRMS results). Even the physical–chemical properties of the solvent (i.e., polarity, dielectric constant, viscosity) are definitory for the yield of extraction, their effects can be augmented or diminished through the interaction with other factors that describe the extractive process. For example, in classic extractive methods (i.e., maceration, infusion, decoction), the solvent exerts the main influence on extraction yield through the physical and chemical interactions with the extracted matrices, its features being those that limit processes like diffusion and partition of analytes from extracted matrices [17], [42]. Conversely, in modern extraction techniques, extraction yield can be considered a consequence of the interaction between solvent and some specific features of the method.

Hence, our attention was further focused on studying the two statistical significant interactions identified in the model, namely between solvent and the other two working parameters, time and ultrasound amplitude, respectively. The presence of two mentioned interactions suggest the appearance of a synergistic effect regarding the TPC extraction yield, when increasing both working parameters at the same time. A great advantage of using a DoE approach, being highlighted in this situation – the DoE not only enhances the optimisation strategy by allowing the identification of interactions, but also allows the quantification of their effects.

#### 3.2.1 Design space and process optimization

The described approach provided an in-depth understanding of the variables’ influence over the process. Further, based on the initial investigation domain and the DoE model, the *Modde* optimisation function was used to generate a design space by introducing the desired TPC values (minimum 39 mg GAE/mL and a target value of 40.5 mg GAE/mL) namely trying to maximize the extraction capacities; our findings were set taking into account that the values of TPC obtained from experimental runs varied from 27.62 to 40.71 mg GAE/mL (Table 1). As previously mentioned, it was established that for the chosen conditions, the optimal ethanolic concentration of the solvent must be 50%, aiming to obtain the best recovery of phenolic compounds from TC aerial parts with a minimum exposure time and applying the lowest amplitude possible. Predicted and experimentally measured values of the TPC extraction, as well as the parameters that describe design space are presented in Table 3*.*

**Table 3:** Predicted and experimentally measured values of the TPC extraction.

TPC (mg GAE/mL)					Recovery (%)
Objective			DoE predicted	Experimentally measured	Predicted vs. measured value
Minimum	Target	Maximum			
39.00	40.50	42.00	40.39	41.04	101.6

**Extraction parameter**	**Value**

Exposure time (min)	6.5
Ultrasounds amplitude (%)	34.8
Ethanolic concentration (%)	50

Although UAE is promoted as an efficient method for obtaining high-quality extracts rich in bioactive compounds, it is well-known that inadequate extraction conditions can lead to their incomplete recovery; subsequently, undesired effects like degradation of interest compounds, formation of secondary products that can interfere with extraction or/and separation of analytes or equipment overloading can occur after an inappropriate UAE [11], [14], [43]. Moreover, it was observed that extraction yield is not proportional with the increasing of extraction parameters' values, it will increase in a dependent-manner until reaching a steady-state which remains constant or decrease, so after that maximum peak, every change regarding extraction parameters values being in fact energy loss [14]. The DoE approach allowed us to establish the optimal values of the afore-mentioned parameters (exposure time – 6.5 min, ultrasounds amplitude – 34.8%) and also to predict the TPC value (40.39 mg GAE/mL) for the extract obtained using the optimal conditions (OpTC). We confirmed the predicted data by applying experimentally the parameters above and measuring TPC for the optimized extract, obtaining a TPC value of 41.04 mg GAE/mL. Our findings fall within the narrow range of acceptable values (39–42 mg GAE/mL), showing a recovery of 101.6%.

**Fig. 5:**
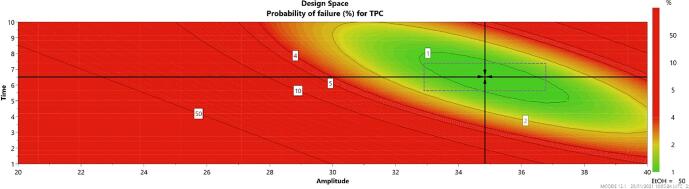
Design space and prediction of failure for TPC.

Based on the mentioned set of constraints, the design space has also been calculated and plotted in Fig. 5. The crosshair that appears in the green area of the design space figure represents the *robust setpoint*, highlighting the input values which will provide the best statistical prediction. The main difference between the robust and optimal setpoints is that the above-mentioned optimal setpoint is focused on finding a solution as close to the target as possible [27]. In the present case, the robust setpoint is overlapped with the optimal setpoint, indicating a highly performant model (design space hypercurbe being described by time values between 5.6 and 7.4 min and amplitudes between 32.9 and 36.8%).

### 3.3 Total phenolic content (TPC) and total flavonoidic content (TFC)

As we previously mentioned, the TPC was used as a quality index for developing an optimized extraction method and for the characterization of the optimized extract (OpTC) (Table 4). This parameter offers a preliminary overview regarding the phytochemical profile of the extracts, their high values being usually correlated with an important antioxidant potential or other bioactivities [44]. *Thymus* species are well-established sources of polyphenols, including flavonoids, but there is a lack of knowledge regarding phenolic content and the profile of TC. Mărculescu et al. [7] reported a TFC value of 0.448%, the result being expressed as rutoside per 100 g of dried herbal drug. Due to the mismatch of the ways that TFC and TPC were expressed, it is difficult to compare our results with the previously obtained ones; still, our preliminary assessments allowed us to estimate the distribution of main types of phenolic compounds in OpTC, concluding that only 21.41% of them are flavonoids. Based on these findings, we guided further steps of the phytochemical analysis to make an in-depth and more comprehensive characterization of OpTC and focus our attention both on flavonoids and other classes of phenolics (i.e., phenolic acids, anthocyanins, stilbenes, etc.).

**Table 4:** Overview of TPC, TFC and antioxidant capacity values measured for OpTC extract.

Assay	OpTC
TPC (mg GA eq./g dw)	156.20 ± 1.32
TFC (mg Q eq./g dw)	33.45 ± 0.36
TEAC (mg TE/g dw)	149.93 ± 0.74
FRAP (mg TE/g dw)	527.35 ± 27.42
DPPH (mg TE/g dw)	79.28 ± 0.41
TBARS (μg/mL)	11.85 ± 0.03
OxHLIA	
Δt = 60 min	10.2 ± 0.3
Δt = 120 min	23.6 ± 0.4

### 3.4 Untargeted phenolic profile of OpTC

In this work, the UHPLC-HRMS phenolic profiling allowed to putatively annotate 246 compounds that were reported in supplementary material considering their relative abundance values, isotopic MS, and MS/MS spectra. Flavonoids represented the most abundant class, consisting of 43 anthocyanins, 63 flavones and derivatives, 44 flavonols, and 21 flavan-3-ols. Besides, other non-flavonoid phenolic compounds were found, namely lignans (11 compounds), lower-molecular-weight phenolics (27 compounds), phenolic acids (32 compounds), and stilbenes (5 compounds). Interestingly, 76 phenolics (31%) were also structurally confirmed, with flavones and derivatives presenting the highest number of MS/MS confirmation against the comprehensive Food Database. Among the most abundant compounds, we found delphinidin, petunidin, and cyanidin 3-glucoside (for anthocyanins), luteolin 7-galactoside, sakuranetin, and cirsilineol (for flavones and derivatives), 3′,4′-dimethylquercetin and 3-methoxynobilitin (for flavonols), isomeric forms of salvianolic acid, lithospermic acid, and phlorin (for other phenolics), and 4-hydroxybenzoic acid, benzoic acid, and phenylacetic acid (for phenolic acids). As the next step, the annotated phenolics were quantified according to pure standard compounds representing the seven classes considered. Overall, other phenolics (quantified as oleuropein equivalents) and flavones (quantified as luteolin equivalents) showed the highest cumulative values, being 796.85 and 793.99 μg/g, respectively. Besides, other particularly abundant classes were represented by flavonols (205.66 μg/g), phenolic acids (98.51 μg/g), and anthocyanins (94.34 μg/g). Finally, a similar content of flavan-3-ols and stilbenes was recorded, being 40.35 and 45.91 μg/g, respectively. As previously reported, the OpTC extract was found to be a rich source of salvianolic acid derivatives (64.27 μg/g), such as salvianolic acid C, D, and L (supplementary material), never described in the scientific literature for this *Thymus* species. Accordingly, salvianolic acids have been recently reported in other *Thymus* species, such as *T. mastichina* [45], *T. vulgaris,* and *T. citriodorus* [46]; besides, salvianolic acid levels in *Thymus* have also been associated with extracts bioactivity, such as anti-proliferative and cytotoxic activity against different cell lines. Interestingly, salvianolic acid C was recently proposed as an inhibitor of SARS-CoV-2 infection, also exerting a potential effect on inhibiting cytokine storm induced by SARS-CoV-2 [47]. Regarding other compounds structurally confirmed by HRMS, we found (among the others) rosmarinic acid, delphinidin, and luteolin 7-glucoside (supplementary material). Rosmarinic acid (belonging to hydroxycinnamic acids) has been previously described as the major phenolic compound in both water and hydroethanolic extracts of *T. citriodorus* and *T. vulgaris* [46]. However, the OpTC extract under investigation revealed a lower content of rosmarinic acid (0.95 μg/g), rather showing a large abundance of hydroxybenzoic acids (such as benzoic acid and 4-hydroxybenzoic acid, on average: 15.41 μg/g). The high abundance of delphinidin (32.12 μg/g) is coherent with the purple-crimson colour of the flower, whilst the presence of luteolin 7-glucoside was also reported in previous work [48] dealing with the analysis of 11 different *Thymus* species.

### 3.5 In vitro biochemical assay of antioxidant potential

One of the most-cited bioactivities of phenolic-rich extracts obtained from plant matrices is the antioxidant potential, the mechanisms of this action being extensively studied [42], [49]. Hence, many *in vitro* methods were developed to evaluate antioxidant activity to achieve an in-depth approach regarding how different phenolic compounds or complexes can act as modulators of oxidative processes [50]. In the present work, we evaluated for the first time the antioxidant potential of a TC phenolic-enriched phytocomplex using 5 complementary biochemical assays (results shown in Table 4). Based on the obtained results, we can consider that OpTC extract exerts a strong *in vitro* antioxidant potential, acting especially as a free-radical scavenger (best results obtained for DPPH assay – 79.28 ± 0.41 mg TE/g). Moreover, results obtained in TBARS assay confirm the ability of OpTC extract to inhibit lipid peroxidation, its activity being comparable or stronger than other *Thymus* extracts (*T. × citriodorus, T. algeriensis*), including the common thyme (*T. vulgaris*) [51], [52], [53]. Similarly, the effectiveness of OpTC as oxidative haemolysis inhibitor was highlighted, the obtained results confirming an inhibitory capacity higher than the positive control (Δ*t_Trolox_* at 60 min being 21.80 ± 0.25 and Δ*t_Trolox_* at 120 min being 43.50 ± 0.82).

### 3.6 Enzyme inhibitory activity

OpTC extract was evaluated for its antiglycosidase, antityrosinase, and anticholinesterase potential, the results being presented in Table 5. As we can observe, the most relevant inhibition rate was described for the antiglucosidase effect (almost 13% from the positive control activity), followed by the anticholinesterase effect; unfortunately, the extract didn't show antityrosinase activity. Taking into account that glucosidase inhibitors are considered promising antidiabetic agents, our preliminary findings encourage future investigations on this extract in order to deeply elucidate its ability to interact with glucose metabolic pathways. This perspective is also supported by previous reports about antidiabetic potential already proven for the extracts obtained from other *Thymus* species like *T. quinquecostatus* Celak (crude extract and its EtOAc fraction), *T. cariensis* (hexane extract), and *T. vulgaris* [54], [55], [56]. However, we reported for the first time the aspects regarding the enzyme-inhibitory potential of TC (including its *in vitro* antidiabetic activity).

**Table 5:** Overview of *in vitro* antioxidant potential of OpTC.

Enzymatic assay		Value
α-Glu (IC_50_, µg/mL)	OpTC	1985.09 ± 84.91
	Acarbose	286.60 ± 36.71
Tyrosinase (IC_50_, µg/mL)	OpTC	N.A
	Kojic acid	50.2 ± 0.15
Acetylcholinesterase (IC_50_, µg/mL)	OpTC	6333.75 ± 411.71
	Galantamine	1.42 ± 0.25

### 3.7 Correlations

**Fig. 6:**
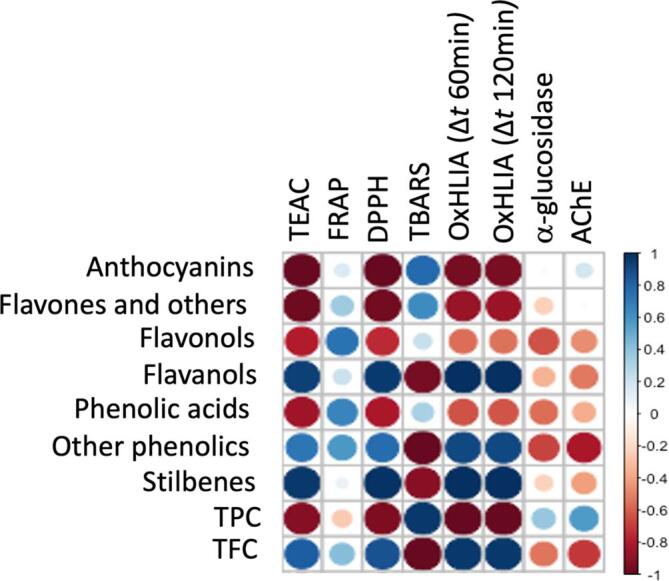
Correlogram considering the significant phenolic classes and the bioactivity values measured.

Pearson's correlation coefficients (r) were then evaluated to unravel those phenolic compounds better correlating with the different bioactivity measured. Overall, the obtained correlogram (Fig. 6) revealed that stilbenes, flavan-3-ols, and other phenolics established the higher number (n = 5) of significant (*p* < 0.05) correlations with the studied bioactivities, followed by flavone derivatives (n = 4), anthocyanins (n = 3), flavonols and phenolic acids (n = 2). Interestingly, other phenolics (including mainly salvianolic acid derivatives) were highly correlated with OxHLIA values, both at 60 (r = 0.90) and 120 min (r = 0.91), while lower r values were observed for TEAC, FRAP, and DPPH values (supplementary material). The high correlation values observed between other phenolics and antioxidant capacity values are coherent with the strong and negative (*p* < 0.05; r = −1.00) correlation coefficients measured towards TBARS assay (Fig. 6. The protection of phenolics (such as salvianolic acids) against oxidative stress was also confirmed by Liu et al. [57], reporting the inhibition of malondialdehyde (MDA) generation, one of the final products of polyunsaturated fatty acids peroxidation in the cells, directly correlated with increased oxidative stress caused by free radicals causes. The same trend in terms of antioxidant potential was also observed for other two phenolic classes, such as stilbenes and flavan-3-ols (Fig. 6. Notably, the HRMS approach highlighted pinosylvin as the most abundant compound within the stilbenes class; this compound has been previously reported [58] as involved in the protection against oxidative stress through the induction of heme oxygenase-1 in human retinal pigment epithelial cells, and having different other *in vitro* activities, such as ORAC, ABTS, and FRAP [59].

## 4 Conclusions

We developed an optimized ultrasound-assisted extraction method for non-volatile polyphenol fraction contained in the aerial parts of *Thymus comosus* Heuff. ex Griseb. et Schenk, taking into account the influence of three extraction parameters (independent variables) – time, ultrasound amplitude and ethanol concentration – on the total phenolic content (dependent variable) of the optimized extract (OpTC). DoE approach allowed us to find the optimal working conditions (exposure time – 6.5 min, ultrasounds amplitude – 34.8% and 50% ethanol solvent concentration) and also to study the interactions between independent variables and their relevance on the quality of the extract. Moreover, we applied an UHPLC-HRMS method for the phenolic profiling of OpTC, revealing a high content of flavonoid-type compounds. The presence of different classes of bioactive compounds was correlated for the first time with the antioxidant and enzyme inhibitory activities of the extracts obtained from this species, highlighting its importance as potential source of bioactive compounds. Additionally, our work was focused to bring several new insights regarding the link between phytochemical profile and bioactive potential of *T. comosus*; we emphasized for the first time the presence of several secondary metabolites with proven health benefits (i.e. anthocyanin derivatives, salvianolic acids) in the extracts obtained from this species and their enzyme-inhibitory activity, which can be considered as a main element of originality for the present study.

## CRediT authorship contribution statement

**Mihai Babotă:** Conceptualization, Methodology, Software, Writing – review & editing. **Oleg Frumuzachi:** . **Alexandru Gâvan:** Conceptualization, Methodology, Software, Validation. **Cristian Iacoviță:** Visualization, Investigation. **José Pinela:** Conceptualization, Methodology, Software. **Lillian Barros:** Conceptualization, Methodology, Software. **Isabel C.F.R. Ferreira:** Supervision. **Leilei Zhang:** Visualization, Investigation. **Luigi Lucini:** Software, Validation. **Gabriele Rocchetti:** Supervision. **Corneliu Tanase:** Data curation, Writing – original draft. **Gianina Crișan:** Supervision. **Andrei Mocan:** Conceptualization, Methodology, Software, Funding acquisition.

## Declaration of Competing Interest

The authors declare that they have no known competing financial interests or personal relationships that could have appeared to influence the work reported in this paper.
